# Neutron activation analysis of manganese in samples of human sternum

**DOI:** 10.1007/s10967-025-10574-5

**Published:** 2025-11-26

**Authors:** Song Yue, Elizabeth Helen Jaye, Gill Nelson, Brad A. Racette, Linda H. Nie

**Affiliations:** 1 School of Health Sciences, Purdue University, West Lafayette, IN United States of America; 2 Department of Radiation Oncology, University of Florida, Gainesville, FL United States of America; 3 Department of Radiation Oncology, The Ohio State University, Columbus, OH United States of America; 4 Barrow Neurological Institute, Phoenix, AZ United States of America; 5 Faculty of Health Sciences, School of Public Health, University of the Witwatersrand, Johannesburg, South Africa

**Keywords:** IVNAA, DD neutron generator, Manganese (Mn) overexposure and toxicity

## Abstract

A compact neutron activation analysis (NAA) system utilizing a deuterium-deuterium (DD) neutron generator was developed to detect manganese (Mn) in bone in vivo. Mn was selected for study due to the elevated risk of overexposure among certain occupational groups and segments of the general population, which can result in irreversible neurotoxic effects. To improve the accuracy of DD neutron generator-based in vivo NAA (IVNAA) system, 12 human cadaver bone samples were analyzed. The system was evaluated, and good agreement was observed between its results and the conventional reactor-based NAA across six irradiated samples. The remaining samples, however, showed discrepancies—likely due to presence of residual soft tissue or the fact that some of the samples are not really bones. A major limitation in analyzing these samples was the lack of calcium (Ca) signals, which prevented Mn/Ca normalization, a critical step for correcting geometric and mass-related variations. These results emphasize the importance of proper sample preparation and verification when conducting ex vivo measurements with the laboratory system. In contrast, in vivo NAA is less affected by such inconsistencies due to a more consistent bone composition and hence the ability to normalize Mn levels to Ca. Overall, accurate application of NAA in environmental and health research requires careful consideration of sample integrity and appropriate normalization methods.

## Introduction

Manganese (Mn) is an essential element in human body [1]. It is a transition metal that serves as structural and catalytic cofactor in proteins and enzymes. For example, Mn maintains immune cells and metabolism of lipids [2]. Usually, health problems related to Mn deficiency is rarely reported. This is because dietary Mn intake can meet the demand. However, its overexposure has raised concerns for both occupational and general populations. Workers, such as miners and welders, have high possibility of being overexposed to Mn [3–5]. Environmental exposures to Mn occur from industrial emissions, combustion engines, and specific pesticides [5–8]. Mn has been shown to cause damage to the nervous system [9–12], with its harmful effects often being irreversible.

Thus, accurately measuring Mn for early risk assessment is crucial. At the same time, identifying an appropriate biomarker for Mn quantification is equally important. Toenail, urine, hair, and blood samples used as indicators of Mn levels are susceptible to external contamination and can be influenced by recent human activities. In addition, these biomarkers only reflect recent Mn exposures. One potential reliable biomarker for long-term Mn exposure reported in the literature is bone, which contains over 30% of total body Mn [13]. However, measuring Mn in human bone is challenging. Current computer tomography (CT) and magnetic resonance imaging (MRI) can only take anatomical pictures of tissues. Neither of them can obtain elemental information from bone. Neutrons, on the other hand, can interact with atomic nucleus directly regardless of storage organ and element type. Based on this unique feature, in vivo neutron activation analysis (IVNAA) has been developed to measure elemental composition [14–18]. Regarding neutron source, compact neutron generator offer significant advantages over accelerator-driven or reactor-based neutron facilities, given its affordable cost and great portability.

Previously, our lab tested the feasibility of a DD neutron generator based IVNAA system for Mn quantification [19, 20]. We also utilized the system for quantification of other metals and trace elements including aluminum, sodium, and potassium in human and animal tissues [21–23]. In this study, we aimed to compare the Mn measurement results in human cadaver bone obtained using reactor-based irradiation with those obtained using a neutron generator-based IVNAA system, which may provide insights for improving the accuracy of the latter method.

## Material and methods

Utilizing neutrons to quantify Mn requires thermal neutron capture reaction (13.4 barn, $1{\text{ barn}} = 10^{ - 24} {\mathrm{cm}}^{2}$), which activates ^55^Mn and the resulted radioisotope (^56^Mn) releases 847 keV characteristic gamma ray for signal acquisition. This physical process is described in Eq. 1. The count number of 847 keV gamma-ray collected by the detector after neutron irradiation can be theoretically calculated by Eq. 2.

1 $$ \begin{array}{*{20}c} {{}_{ }^{55} Mn + n \to {}_{ }^{56} Mn + \gamma } \\ \end{array} $$

2 $$ \begin{array}{*{20}c} {S = R \times N_{0} \times \varepsilon \times \theta \times F_{n} \times \left( {1 - e^{{ - \lambda t_{i} }} } \right) \times e^{{ - \lambda t_{d} }} \times \frac{{\left( {1 - e^{{ - \lambda t_{c} }} } \right)}}{\lambda }} \\ \end{array} $$

where $R$ is the reaction rate, which is an integration of neutron flux multiplied by thermal neutron cross section over the entire neutron energy spectrum; $\lambda$ is the decay constant; ${N}_{0}$ is the number for the target nuclide; $\varepsilon$ is the detector efficiency; $\theta$ is the branch ratio of the characteristic gamma ray for specific radionuclides produced; ${F}_{n}$ is the neutron flux; ${t}_{i}$ is irradiation time; ${t}_{d}$ is decay time; and ${t}_{c}$ is counting time.

Two neutron sources were used in this comparative study: our transportable DD neutron generator and Purdue University Reactor Number One (PUR-1).

### Human cadaver bone samples

**Fig. 1:**
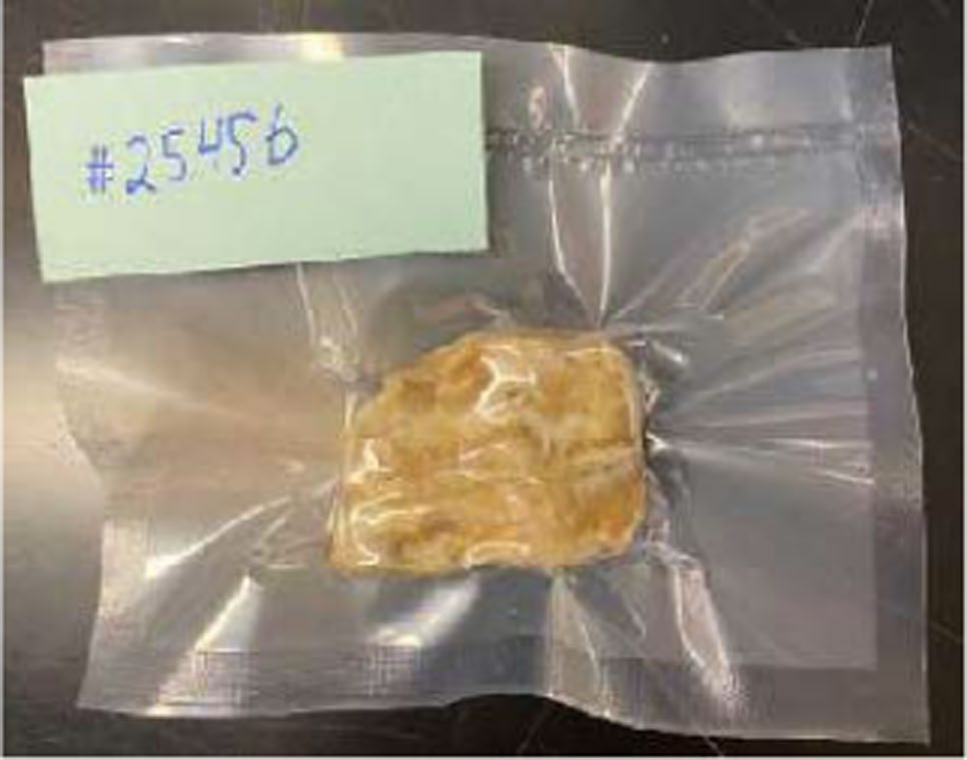
Human sternum sample #25456

Sternal tissue for this study was collected as part of a study investigating the neuropathology of occupational Mn exposure in Mn-exposed miners and control miners. The University of the Witwatersrand Human Research Ethics Committee (clearance certificate no. M2011151) approved this study. Next-of-kin provided informed consent for autopsy, including removal of a portion of sternum. Four of the miners were Mn miners while the other eight were asbestos miners. Each sample was given an ID number. The samples vary in size and in the extent to which soft tissue was removed. Some samples have a clear bone-like appearance, while others do not. All samples were contained in Formalin jars stored in a refrigerator to prevent decomposition. In order to irradiate and measure the samples, they were removed from the Formalin, dried with paper towels, and then vacuum sealed in plastic bags. Following irradiation and measurement, the samples were placed back in the Formalin jars. For the reactor NAA, samples were cut to small pieces, and for the lab NAA, samples were irradiated as a whole. An example of one of the vacuum sealed bones is shown in Fig. 1.

### Reactor irradiation experiment design

Reactor is considered a very useful NAA research resource for environmental and biomedical study [24]. PUR-1 is a stable, low-power reactor neutron source moderated and shielded by water and concrete. The uniform distribution of thermal neutrons provides consistent irradiation conditions. To determine the thermal neutron flux, separate gold foil (Au) was attached to bone pieces, which were cut off from different cadaver bone samples. For irradiation, they were delivered to same location near the reactor core using a separate pipe. The Au mass was measured in advance for parameter ${N}_{0}$ in Eq. 2. The reaction rate R is replaced by thermal neutron capture cross section $\sigma$ of target Au atoms given much higher thermal neutron flux in reactor. Other parameters, including${t}_{i}$,${t}_{d}$, and ${t}_{c}$, were recorded accordingly.

Two methods were applied independently to calculate the Mn concentration in reactor irradiation: a) One method involves the absolute calculation based on Eq. 2, where the thermal neutron flux is determined through Au irradiation. b) The other method employs relative counting with a Mn standard made of $Mn{Cl}_{2}*4{H}_{2}O$. The characteristic gamma ray counts were normalized to 5 min decay and 1 h counting using Eq. 2. The irradiation time in reactor for all experiments was fixed at 30 min. A high efficiency High Purity Germanium (HPGe) detector located in the reactor lab was used for gamma ray detection. The detector’s efficiency was calibrated using a multi-nuclide standard source.

**Fig. 2:**
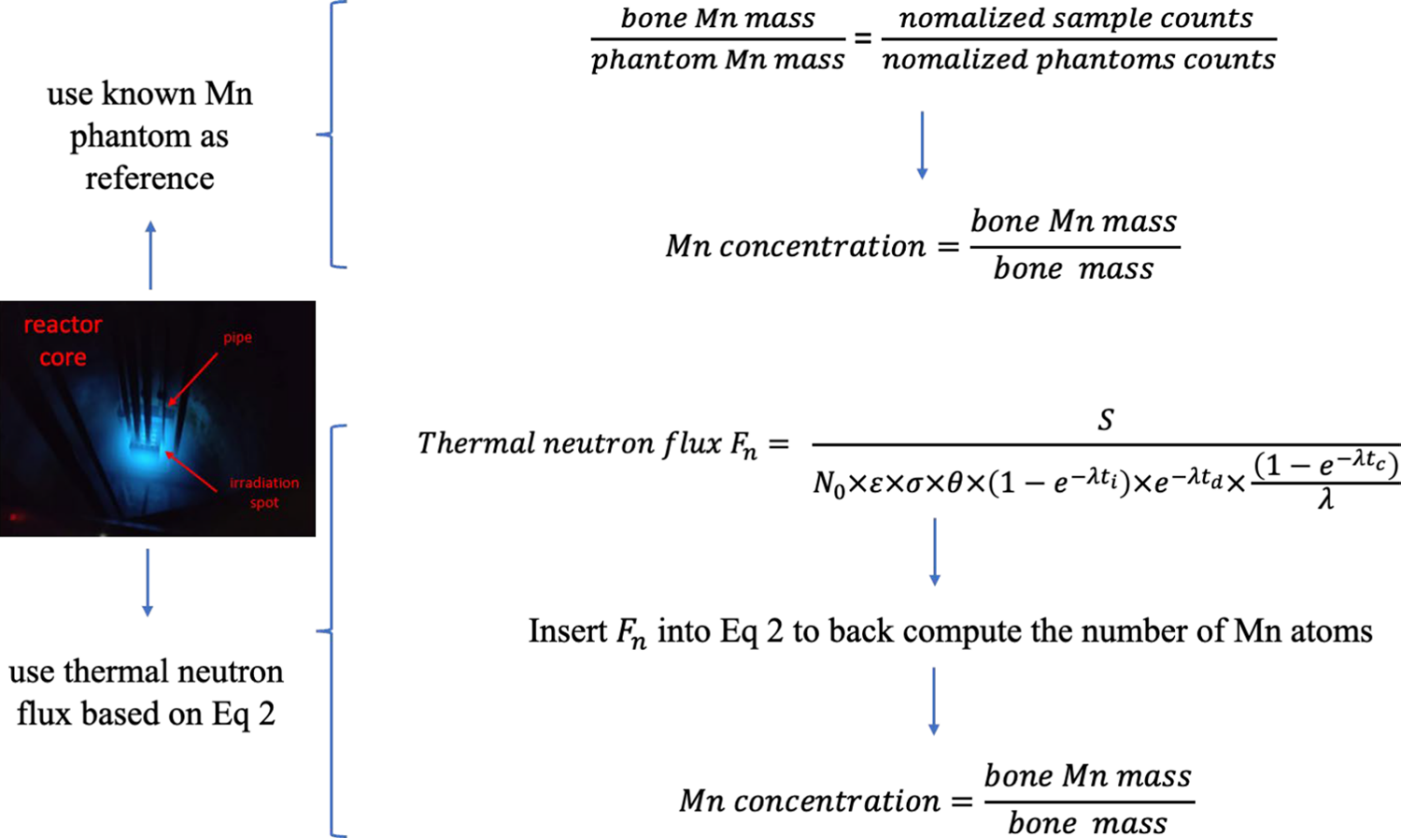
Calculation workflow for reactor irradiation

All the constants that are related to Mn concentration determination using reactor NAA were summarized in Table 1 and Table 2. For clear illustration, the relevant details were shown in Fig. 2.

**Table 1:** Constants for Mn calculations

Parameters of Mn in Eq. 2	Value
thermal neutron capture cross section $\sigma$	13.4 barn
branch ratio $\theta$	98.9%
half life	2.579 h

**Table 2:** Constants of Au for thermal neutron flux calculations

Parameters of Au in Eq. 2	Value
thermal neutron capture cross section $\sigma$	98.8 barn
branch ratio $\theta$	100%
half life	2.695 days
number of Au atoms $N_{0}$	$\frac{{m\left( g \right)}}{{M\left( {g/{\mathrm{mol}}} \right)}} \times {\text{Avogadro constant}}$

### Laboratory NAA system

**Fig. 3:**
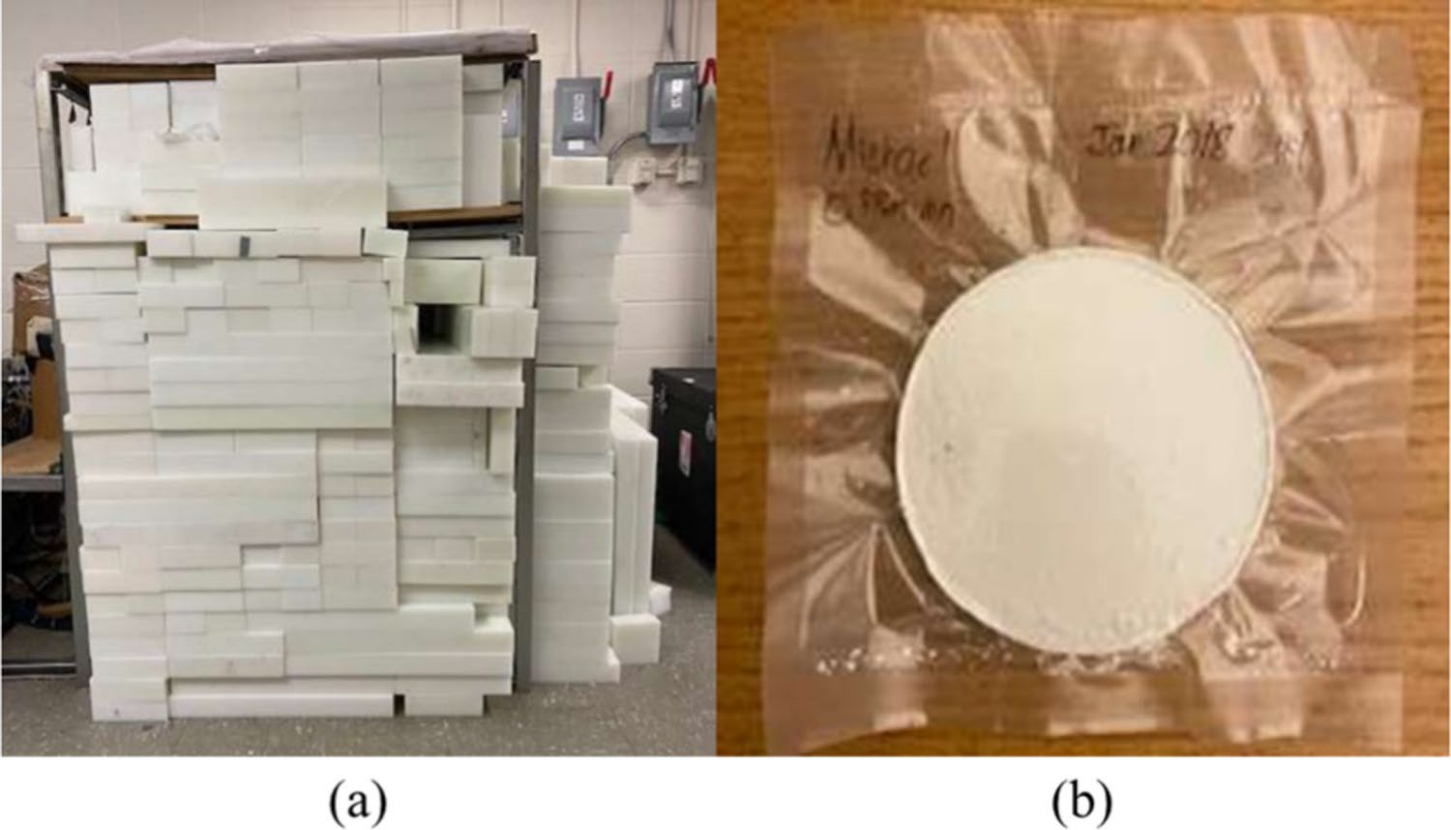
**a** DD neutron generator **b** a bone equivalent Mn phantom

The high energy neutrons (2.45 MeV) from our well-shielded DD neutron generator (Fig. 3a) were moderated to thermal energy levels (around 0.025 eV) using carefully chosen moderator materials. The irradiation cave construction and relevant radiation dose calculations were documented in the reference [20]. Cadaver bone samples were irradiated for 10 min, followed by a 5-min decay, and then transferred to a HPGe detector located in the DD neutron generator lab for a one-hour gamma ray measurement. The detector efficiency was determined using a multi-nuclide source.

To facilitate in vivo measurement, the system needs to be calibrated by a series of human bone-equivalent phantoms with various Mn concentrations and density of $1.85g/{cm}^{3}$ (Fig. 3b), which were irradiated following the same time scheme as the bone samples. These standard phantoms were fabricated based on bone compositions of a reference man recommended by International Commission on Radiological Protection [25]. The chemical compounds in the phantoms include ${CaSO}_{4}$, ${NH}_{4}Cl$, ${NaNO}_{3}$, ${MgSO}_{4}$ and ${Mn({NO}_{3})}_{2}$. The detailed calculations and phantom making procedures can be found in our previous paper [19]. Equation 3 was used to correct the mass differences between the phantoms and the bone samples.

3 $$ \begin{array}{*{20}c} {{\text{Mn concetration}} = \frac{{\text{Mn counts}}}{{\text{calibration line slope}}} \times \frac{{\text{phantom mass}}}{{\text{ bone sample mass}}}} \\ \end{array} $$

## Results

### Detector and IVNAA system calibration

The two HPGe detectors (GMX 100P4-95-A and GEM 20P4-70) were calibrated by multi-nuclide source independently. The irradiated bone samples in reactor and lab were measured using corresponding detectors. Both detectors and their software for gamma ray spectra collection are from ORTEK (AMETEK, Oak Ridge, TN).

**Fig. 4:**
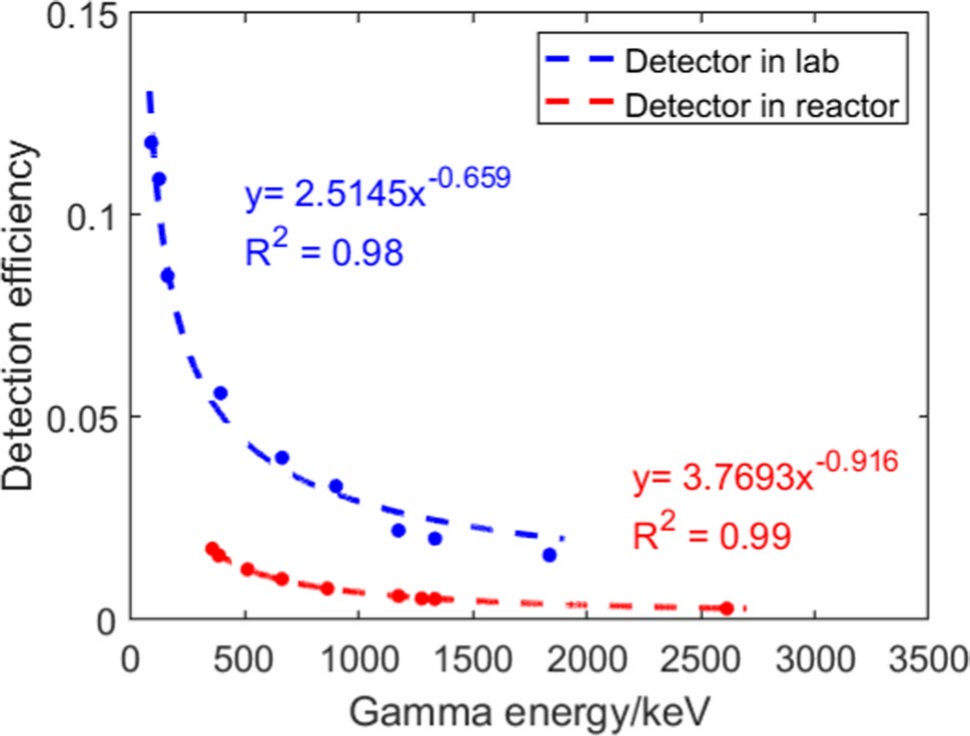
Efficiency curves for two used HPGe detectors

The two HPGe detectors are the similar except for their head surface, hence the detection efficiency. Counting efficiency curves of the two detectors used for laboratory NAA system and reactor irradiation measurements were shown in Fig. 4. When conducting experiments, the samples were placed in the same location as the multi-nuclide source that was used for calibration to reduce uncertainties.

### Mn concentration from reactor experiments

**Fig. 5:**
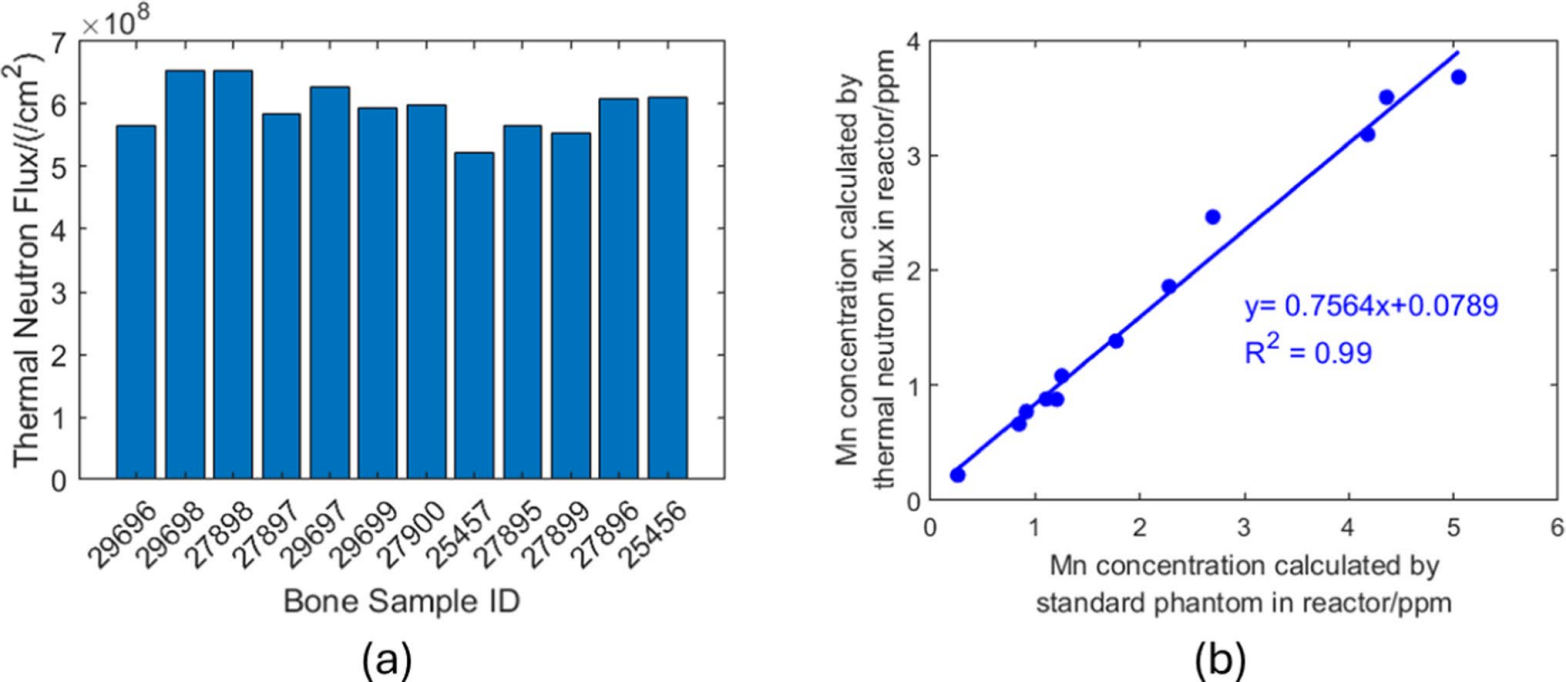
Mn calculations in reactor **a** thermal neutron flux when irradiating each bone piece **b** Mn concentration comparison between two methods

In absolute method, the number of Mn atoms and hence the Mn concentration is calculated by absolute thermal neutron flux, which was determined by Au foil. The derived neutron flux was shown in Fig. 5a. In relative method, Mn concentration was calculated by using the ratio of the counts obtained from the sample to that obtained from the standard Mn reference material. Figure 5b plots the correlation between the Mn concentrations calculated by the two methods.

Overall, the results obtained from two methods reached good agreement. From Fig. 5a, the thermal neutron flux fluctuated for different irradiations. However, the corresponding flux was used for each sample calculation. Its effect can be removed. The discrepancy in the two methods could be resulted from the counting efficiency differences between samples and the standard multiple nuclide source. More explanations are provided in the discussion section.

### Mn concentration from laboratory NAA system

**Fig. 6:**
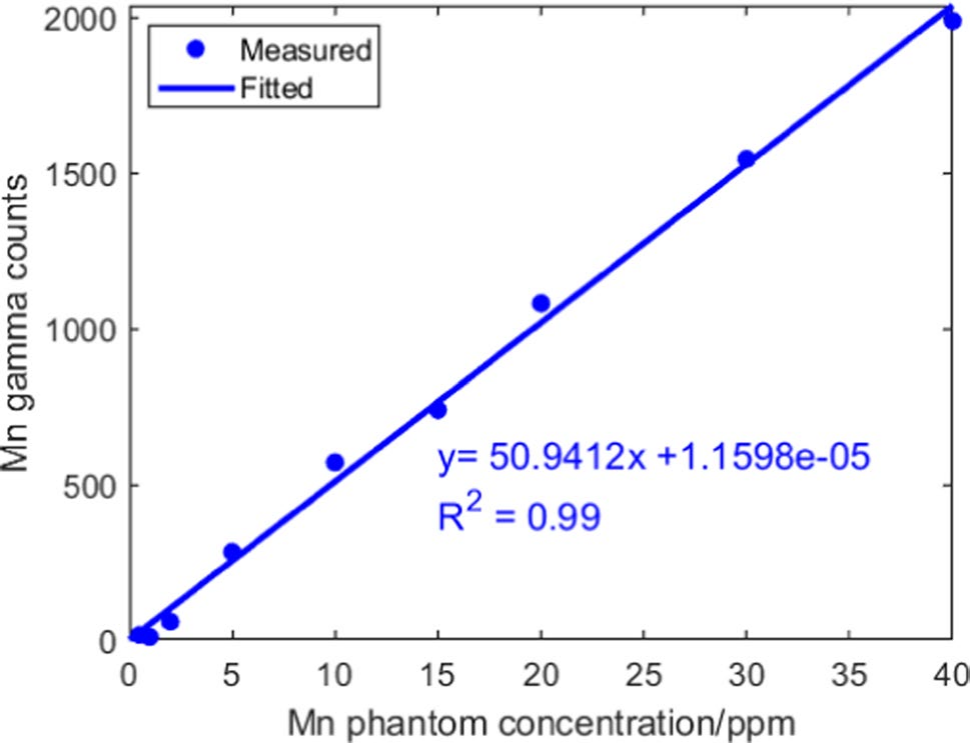
Mn calibration line for laboratory NAA system

The phantoms used for laboratory NAA system calibration were doped with 0, 0.5, 1, 2, 5, 10, 15, 20, 30 and 40 ppm Mn. The absolute gamma counts versus Mn concentration calibration line was shown in Fig. 6.

**Fig. 7:**
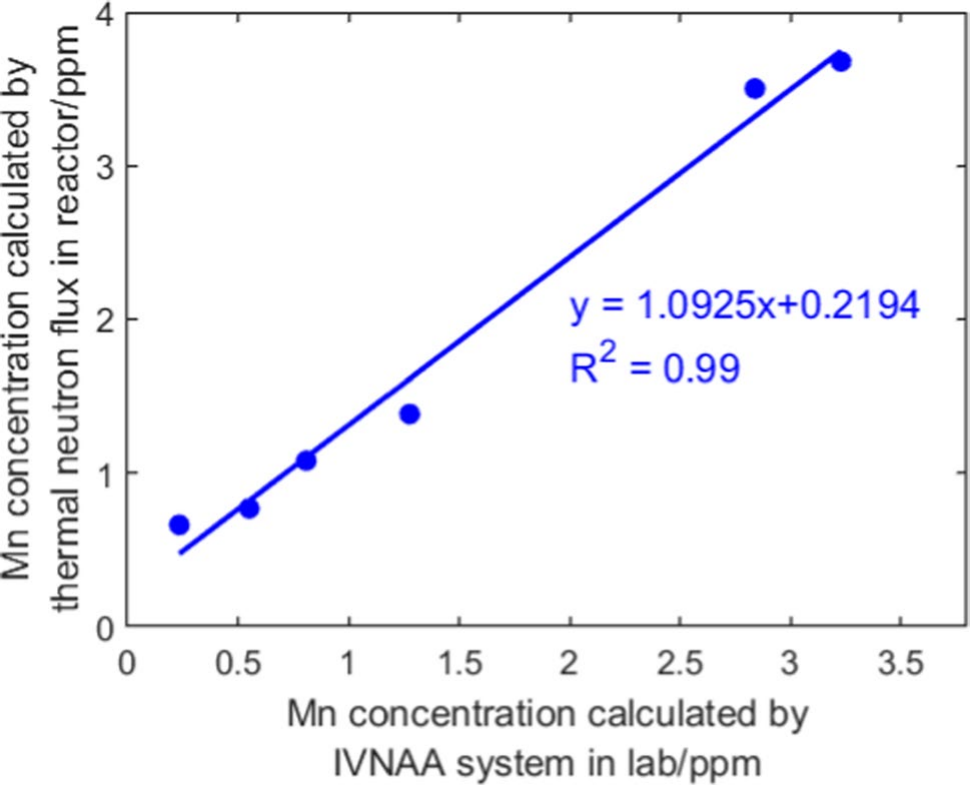
Mn concentrations calculated by laboratory NAA versus reactor NAA system for selected samples

**Fig. 8:**
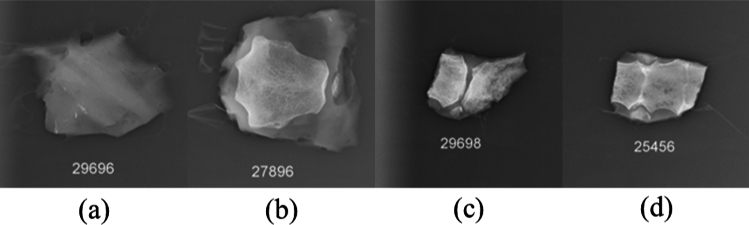
X-ray images for composition. **a** shows the sample doesn’t contain bone, primary cartilage or soft tissue. **b** shows the sample partially contains bone. **c** and **d** show samples are primarily bone

Instead of the cut-off pieces, the whole cadaver bone samples were irradiated in the irradiation cave. The calculated Mn concentrations of 6 cut-off pieces using Eq. 3 were compared with the results based on thermal neutron flux calculations, as shown in Fig. 7. The Mn concentrations obtained by laboratory NAA system matched with the results from the reactor NAA for half of the samples, where a strong correlation was observed in 6 samples. The other 6 cut-off pieces were eliminated from the results because of large discrepancy between the cut-off and the whole sample. There are 2 potential explanations for the exclusion of the six outliers. First, it can be interpreted by much larger sample size used in lab irradiation. The whole sample contained more tissues other than human bone, and it contributed to higher sample mass. In addition, it can be attributed to composition differences between cut-off pieces in reactor and whole bone sample in lab irradiation experiments. When investigating this problem, we found that these samples contained zero or very small portion of Calcium (Ca), which indicated they were not human bone. Most of the samples include soft tissue and cartilage, and some lack bone entirely. X-ray imaging was used to illustrate these differences, revealing significant variation in sample composition (Fig. 8). The two factors introduced inconsistent calculations and incomplete corrections.

## Discussion

We compared Mn measurements in human bone, utilizing reactor and lab NAA. In reactor NAA, calculations were based on thermal neutron flux, which was determined by gold foil, and standard Mn reference material. A linear regression was clearly observed (Fig. 5b), with a slope of 0.75. This deviation of the slope from 1 can be attributed to geometry differences between the samples and the standard multiple nuclide source used for efficiency calibration. Specifically, the experimental efficiency determined using the Mn reference material was 0.59%, whereas the efficiency calculated from the efficiency curve was 0.78%. The use of this calculated efficiency in the concentration determination accounts for the observed slope of 0.75.

It is reasonable to consider the potential contribution of fast and epithermal neutrons to manganese (Mn) activation and subsequent gamma-ray emission. However, the influence of non-thermal neutrons on Mn activation is negligible and can be excluded without compromising the accuracy of the results. Empirical neutron irradiation experiments employing gold foil activation measurements confirm that approximately 99% of the observed gamma-ray emissions arise from Mn nuclei activated by thermal neutrons.

Compared to reactor NAA, the laboratory NAA irradiated the whole bone sample. Extra human tissues were attached to them and added to the deviation of the results. Moreover, since only the mass factor was corrected using Eq. 3, the geometry differences between bone equivalent phantoms and bone samples were not accounted. The geometry correction for bone phantoms can be conducted by normalizing Mn to Ca using the Mn/Ca ratio as done in our previous papers [19]. However, as discussed above, most of the samples include soft tissue and cartilage and several samples don’t contain any bone. In this case, Mn/Ca normalization is not applicable. This indicates the importance of verifying sample composition and collection procedures prior to conducting NAA experiments. In the laboratory-based in vivo NAA system, variations in sample composition can be addressed by normalizing Mn counts to Ca within the bone, using data from the collected spectrum. This approach involves calculating the Mn/Ca ratio and requires careful selection of target organs for irradiation. However, the accuracy of this method may be slightly compromised in individuals with bone disorders such as osteoporosis, where abnormal calcium levels in the bone could distort the Mn/Ca ratio and lead to inaccurate Mn concentration estimates.

## Conclusions

This comparative study examined several factors affecting human bone Mn measurement, using reactor NAA and DD-neutron generator-based lab NAA. Our results show that it is important to verify the composition and characteristics of the samples when conducting ex vivo measurements with the laboratory system. In contrast, in vivo NAA is less affected by such inconsistencies due to a more consistent bone composition and hence the ability to normalize metal levels such as Mn to Ca.
